# Status of Pandemic Influenza Vaccination and Factors Affecting It in Pregnant Women in Kahramanmaras, an Eastern Mediterranean City of Turkey

**DOI:** 10.1371/journal.pone.0014177

**Published:** 2010-12-01

**Authors:** Ali Ozer, Deniz Cemgil Arikan, Ekrem Kirecci, Hasan Cetin Ekerbicer

**Affiliations:** 1 Department of Public Health, Medical Faculty, Kahramanmaras Sutcuimam University, Kahramanmaras, Turkey; 2 Department of Obstetrics and Gynecology, Medical Faculty, Kahramanmaras Sutcuimam University, Kahramanmaras, Turkey; 3 School of Health, Kahramanmaras Sutcuimam University, Kahramanmaras, Turkey; The University of Hong Kong, Hong Kong

## Abstract

**Background:**

Pregnant women are a target group for receipt of influenza vaccine because there appears to be an elevated mortality and morbidity rate associated with influenza virus infection in pregnant women. The goal of this study is to determine the factors affecting the decisions of pregnant women in Turkey to be vaccinated or not for 2009 H1N1 influenza.

**Methodology:**

We enrolled 314 of 522 (60.2%) pregnant women who attended to the antenatal clinics of the Medical Faculty of Kahramanmaras Sutcuimam University's Department of Gynecology and Obstetrics between December 23, 2009, and February 1, 2010. We developed a 48-question survey which was completed in a face-to-face interview at the clinic with each pregnant woman.

**Principal Findings:**

Of the 314 pregnant women, 27.4% were in the first trimester, 33.8% were in the second trimester, and 38.8% were in the third trimester. Twenty-eight pregnant women (8.9%) got vaccinated. Of all the women interviewed, 68.5% stated that they were comfortable with their decisions about the vaccine, 7.3% stated they were not comfortable, and 24.2% stated that they were hesitant about their decisions. The probability of receiving the 2009 H1N1 vaccine was 3.46 times higher among working women than housewives, 1.85 times higher among women who have a child than those who do not, and 1.29 times higher among women with a high-school education or higher than those with only a secondary-school education and below. Correct knowledge about the minimal risks associated with receipt of influenza vaccine were associated with a significant increase in the probability of receiving the 2009 H1N1 vaccine.

**Conclusions/Significance:**

The number of pregnant women in the study group who received the 2009 H1N1 vaccine was very low (8.9%) and two-thirds of them stated that they were comfortable with their decisions concerning the vaccine. Our results may have implications for public health measures to increase the currently low vaccination rate among pregnant women. Further studies are required to confirm whether our findings generalize to other influenza seasons and other settings.

## Introduction

2009 H1N1 has been identified as the cause of a widespread outbreak of febrile respiratory infection worldwide [1]. In both seasonal influenza epidemics [2], [3] and previous pandemics [4], [5], [6], the mortality and morbidity rate from influenza infection was higher in pregnant women than in non-pregnant women. Mechanical, immunologic, and hormonal changes in pregnancy contribute to this increased risk [3], [7], [8]. More pregnant women than non-pregnant women are hospitalized due to acute respiratory diseases and cardiopulmonary cases [2], [3], [9]. The influenza vaccination is the most effective way to protect pregnant women from influenza [10]. The World Health Organization Strategic Advisory Group of Experts, the European Union Health Security Committee and the Early Warning and Response system, and the United States Advisory Committee on Immunization Practices, have all stated that pregnant women should receive precedence in receiving the 2009 H1N1 vaccine [11], [12], [13], [14].

In Turkey, pandemic flu cases have been seen in every city by November 15, 2009. Laboratory examination has determined that 627 people have died due to pandemic flu. Of these, 40 were pregnant or puerperant. On November 2, 2009 the Turkish Ministry of Health announced that all health workers were to receive vaccinations. On November 16, 2009 this vaccination group was expanded to include children under 5 years of age. On December 7, 2009 the Ministry of Health announced that pregnant women could receive vaccinations. And finally, on December 17, 2009 the entire Turkish population could be vaccinated. At this point, the Ministry of Health conducted a free mass vaccination campaign [15]. This push sparked the Turkish media, communities and politicians to start discussing and debating the safety and efficacy of the 2009 H1N1 vaccine.

This study aims to determine the factors affecting the 2009 H1N1 vaccination situation, and the decisions of pregnant women, a pandemic risk group, whether to be vaccinated or not.

## Methods

This study was conducted at the antenatal clinics of the Medical Faculty of Kahramanmaras Sutcuimam University's Department of Gynecology and Obstetrics (Kahramanmaras, Turkey) between December 23, 2009 and February 1, 2010. During that time 522 pregnant women applied to our antenatal clinic, 314 (60.2%) of whom choose to join the study. The remaining 208 (39.8%) refused to be involved in the study. We obtained research ethics approval from the Ethics Committee of Kahramanmaras Sutcuimam University before initiating the study, and collected signed informed consent from all subjects.

We drew information from the literature to develop a 48-question survey. One of our researchers then used this survey to conduct face-to-face interviews at the clinic with each pregnant woman. Ten questions were related to sociodemographic characteristics, 1 to status of the vaccination, 1 to the person who made the decision whether or not to vaccinate, 1 to whether the participant felt comfortable or not with her decision about the vaccination, 10 to the factors affecting the participant deciding whether or not to vaccinate, 15 to “2009 H1N1 vaccine's side effects (HVSE),” three to “2009 H1N1 vaccine's side effects related to pregnancy (HVSERP),” and four to “beliefs about a mass 2009 H1N1 vaccination campaign conspiracy (BMHVCC).” Three questions related to “other attitudes and beliefs related to 2009 H1N1 vaccine (ABRHV).” Each correct answer for questions related to HVSE, HVSERP, BMHVCC, and ABRHV was calculated as one point and the total points in each sub-group were calculated.

### Statistical Analysis

We used the Statistical Package for Social Sciences (SPSS Inc., Chicago, IL, USA), version 15.0, to perform statistical analyses. The data were initially tested for normal distribution via the Kolmogorov-Smirnov test and found to be abnormal (p<0.05). We utilized chi-square and Mann-Whitney U tests to compare among groups. Logistic regression was used to determine the independent effects of the associations between vaccination decision and explanatory variables, estimating the adjusted odds ratio and confidence intervals (95%). A multivariable logistic regression model included variables whose associations with vaccination decisions were statistically significant, defined as p<0.05.

## Results

The average age of the pregnant women in the study was 26.5±5.2 years. Of the total, 27.4% of the women were in the first trimester, 33.8% were in the second trimester, and 38.8% were in the third trimester of pregnancy. Of the 314 pregnant women, 8.9% (28 women) decided to get the vaccine. Based on age groups, education, place of residence, chronic disease situation, and trimester at the time of the survey, there was not any significant difference with regard to vaccination status (p>0.05). However, regarding occupation, housewives received statistically significantly fewer 2009 H1N1 vaccinations than did women of working group (p<0.05) (Table 1).

**Table 1:** The affect of sociodemographic characteristics on the 2009 H1N1 vaccination status of pregnant women.

	Vaccination status		
	Vaccinatedn = 28	Not Vaccinatedn = 286	
Variables	%	%	P*
**Age**			
24 and below	7.4	92.6	
25–29	6.5	93.5	0.14
30 and above	14.1	85.9	
**Education**			
Secondary school and below	7.3	92.7	0.09
High school and above	13.4	86.6	
**Occupation**			
Housewife	6.3	93.7	<0.001
Working	20.3	79.7	
**Place of Residence**			
City centre	7.9	92.1	
District centre	7.9	92.1	0.56
Other	11.8	88.2	
**Number of Children**			
0	7.9	92.1	
1	4.9	95.1	0.04
2	16.3	83.7	
3 and above	5.2	94.8	
**Chronic Disease**			
Yes	8.4	91.6	0.43
No	11.8	88.2	
**Trimester**			
1	9.3	90.7	
2	11.3	88.7	0.44
3	6.6	93.4	

*; Chi-square test.

With respect to spouses, 37.9% of the study group stated that they made their decisions on their own, 10.5% said their vaccination decisions were made by their spouses, and 51.5% said they decided with their spouses. In addition, 68.5% of the pregnant women stated that they were comfortable with the decisions they made concerning the vaccine, 7.3% stated that they were not comfortable, and 24.2% stated that they were hesitant. The decisions of having vaccination were affected by television, health personnel, and suggestions from relatives. The decisions of not vaccinating were affected by television, explanations from the Ministry of Health, and explanations from politicians.

The median HVSE points of the pregnant women who were and were not vaccinated were three and the differences among the groups was statistically insignificant (p>0.05). The median BMHVCC points of the pregnant women who were and were not vaccinated were 1 and the differences among the groups was statistically insignificant (p>0.05). Although the median HVSERP points of pregnant women who were vaccinated were 0.5, the median HVSERP points of pregnant women who were not vaccinated were 0, and this difference was statistically significant (p<0.05) (Table 2).

**Table 2:** The univariate analysis of factors affecting the vaccination status of pregnant women.

	Vaccination status				
	Vaccinatedn = 28		Not Vaccinatedn = 286		
Variables	n	%	n	%	p
**The vaccine protects 100% against the 2009 H1N1**					
Yes	20	9.5	190	90.5	0.59*
No	8	7.7	96	92.3	
**The 2009 H1N1 spreads much faster than the seasonal flu**					
Yes	4	6.1	62	93.9	0.36*
No	24	9.7	224	90.3	
**The fatality of 2009 H1N1 is lower than that of the seasonal flu**					
Yes	24	9.6	227	90.4	0.42*
No	4	6.3	59	93.7	
**HVSE**	(Min.-Med.-Max)0-3-12		(Min.-Med.-Max)0-3-12		0.66**
**HVSERP**	(Min.-Med.-Max)0-0.5-3		(Min.-Med.-Max)0-0-3		0.02**
**BMHVCC**	(Min.-Med.-Max)0-1-4		(Min.-Med.-Max)0-1-4		0.34**

*; Chi-square test.

**; Mann-Whitney U test.

As a result of the applied multiple logistic regression analysis, we calculated the odds ratios of the factors we thought could have contributed to the vaccination decision. The vaccination rate was higher among the pregnant women who were working and who answered correctly the questions related to the HVSERP (Table 3).

**Table 3:** Multiple logistic regression analysis of the independent variables affecting the decision of vaccination of pregnant women.

Factors	Odds Ratio	% 95 Confidence Interval	p value
**HVSERP**	1.34	1.00–1.79	0.04
**Education**			
Secondary school and below	1.00		
High school and above	1.29	0.48–3.49	0.60
**Occupation**			
Housewife	1.00		
Working	3.46	1.33–8.95	0.01
**Having a child**			
No	1.00		
Yes	1.85	0.68–5.01	0.22

In the research group, 62.4% of the participants thought that the 2009 H1N1 vaccine would only be tested in Turkey, 77.4% stated that there are some other countries manipulating the receipt of the 2009 H1N1 vaccine, and 75.5% thought that the vaccine was harmful in the long term. Moreover, 70.1% of the pregnant women believed that the vaccine could cause a miscarriage, 74.2% thought it could cause deformation in their children, and 72.3% were worried that the vaccine could cause infertility (Table S1).

## Discussion

Media broadcasts and discussion programs have brought about social anxiety and hesitation about the safety of the 2009 H1N1 vaccine in Turkey and many other countries [16]. This situation has affected the vaccination rate of pregnant women, as well as all of society [17]. The 2009 H1N1 vaccine is a new vaccination and the entire world is curious about its effectiveness and reliability [18]. Since the vaccine can show adverse effects on a fetus, many pregnant women are unwilling to adopt the recommendations of public health authorities [19].

Many around the world hold different opinions about the vaccine. It was found that 2% of pregnant women [20] in a 2000 Canadian study and 12.8% of pregnant women [21] in an American study had received the seasonal influenza vaccine. In our study, 8.9% of pregnant women (28 women) received the vaccination. By January 1, 2010, the vaccination rate among pregnant women in the United States was 38% [22]. In another study performed in France that was smilar to the study performed in the United States the acceptability of 2009 H1N1 vaccine was 37.9% among pregnant women [23].

There are many factors affecting the decisions of pregnant women in Turkey to receive the 2009 H1N1 vaccination. In our study, 37.9% of the women stated that they decided on their own whether or not to vaccinate, 10.5% said that the decision was made by their spouses, and 51.5% stated that they made the decision with their spouses. In addition, 68.5% of pregnant women stated that they were comfortable with their decisions concerning the vaccine, 7.3% said that they were not comfortable, and 24.2% stated that they were hesitant. The decisions of having vaccination were affected by television, health personnel, and suggestions from relatives. The decisions of not vaccinating were affected by television, explanations from the Ministry of Health, and explanations from politicians. Intensive discussions about the vaccine have taken place in Turkey, and an overall level of anxiety has developed throughout society about this subject. In the beginning, the Ministry of Health persistently suggested that everyone should be vaccinated and also stated that the Prime Minister and President would be vaccinated. However, the Prime Minister strongly implied that the Minister of Health should not speak for him in front of the media. Moreover, the Prime Minister stated that he and his family would definitely not be vaccinated [[24],[25],[26]]. This situation increased the Turkish society's hesitations about the vaccine. Almost daily, television programs discussed 2009 H1N1 and its vaccine, and featured scientists and politicians defending and contradicting its effectiveness and dangers [27], [28]. These types of discussions and debates could have fortified society's hesitation over the vaccination. Moreover, they could cause vaccination rates to remain low. In our study, participants expressed that their decisions whether or not to get the vaccine were mostly affected by television. Our results show that the media plays the biggest role in informing the public about a pandemic. In a study performed by Jones et al., participants cited the internet as the most common source of information concerning 2009 H1N1. Furthermore, the study found that radio, television, health personnel, and friends were the most common information sources after the internet [27].

In a study made by Lau et al., one-fourth of the study group felt that the vaccine was unsafe, one-third believed it to be effective against influenza, and two-thirds stated that the vaccine had been approved through clinical experiments [18]. As seen in Table S1, two-thirds of the pregnant women in the research group stated that the 2009 H1N1 vaccine had not been approved and would only be given (as a trial) to the public in Turkey. Three-fourths of the pregnant women said that there are some other countries manipulating the receipt of the vaccine, believed that the vaccine was harmful in the long term, and stated that vaccine companies were responsible for creating 2009 H1N1. Our results show how fear is developed in society, and how media broadcasts about scientific subjects are primarily accredited for conspiracy theories. Nearly three-fourths of the pregnant women in our study stated that the vaccine could cause miscarriage, deformation in the child, and infertility. In a 2006 study by Yudin et al. on pregnant women, 80% of the participants stated that they believed the influenza vaccine could lead to defects in their children [28]. These results were similar to what we found in our study.

Possible side effects of the 2009 H1N1 vaccine are: ruddiness, sensitivity or swelling at the site of vaccination, headache, muscle and joint aches, fever, nausea, perspiration, chilling, and tremors. Rare adverse effects include: serious allergic reaction, anaphylaxis, neuritis, nephritis, vasculitis, thrombocytopenia, convulsion, encephalomyelitis, and Guillain-Barre Syndrome [15], [29], [30], [31]. With respect to such side effects, one-third of the pregnant women in our study thought that the vaccine could cause ruddiness, swelling, hardness, cyanosis and pain in the region of vaccination, lassitude and fatigue. One-fourth of the participants stated that the vaccine could cause headache, apoplexy and neuropathies, perspiration and tremors, joint and muscle aches, extensive skin reaction, and reduction in tension. Other side effects that our participants less commonly attributed to the vaccine include: shock, pain throughout the nervous system, and bleeding from reduced clotting. Although the media had intensively discussed the adverse effects of the vaccine, the rate of related knowledge in the study group remained low. This could be because the media hype caused public confusion about the truths and falsities related to the vaccine. In a study by Lau et al., half of the research group stated that the adverse effects of the 2009 H1N1 vaccine were very few or none, while 16% claimed the effects to be many or severe, and 29% said that they did not know about possible adverse effects [18]. In our study, 20% of the pregnant women stated that 2009 H1N1 spreads faster than seasonal flu and that its fatality rate was lower than that of seasonal flu. In a similar study, 36% of the study group stated that the fatality speed of pandemic flu was higher than that of seasonal flu and 42% stated that it spread faster [18]. Due to the panicked atmosphere formed throughout society, our study group could have thought that the fatality of pandemic flu was higher than that of seasonal flu.

As is evident in the logistic regression model in Table 3, among the independent variables, the effect of HVSERP points (p = 0.04) and occupation (p = 0.01) was statistically significant on vaccination decision (p<0.05); but the effect of educational status (p = 0.60) and having a child (p = 0.22) were not statistically significant on vaccination decision (p>0.05). The probability of receiving the 2009 H1N1 vaccine was 3.46 times higher among working women than housewives, 1.85 times higher among women who have a child than those who do not, and 1.29 times higher among women with a high-school education or higher than those with only a secondary-school education and below. Every one point increase in the HVSERP score increased the probability of receiving the 2009 H1N1 vaccine by 1.34 times. These data show that knowing the vaccine's side effects about pregnancy significantly increased the vaccination rate among pregnant women. This finding shows that during a pandemic, it is critical to inform at-risk groups about their special condition.

In conclusion, the H1N1 vaccination rate among pregnant women in our study was too low (8.9%). Half of the study group stated that they made their decisions with their spouses whether or not to be vaccinated, and one-third of the group stated that they felt comfortable with their decisions about vaccination. Every one point increase in the HVSERP score increased the probability of receiving the 2009 H1N1 vaccine by 1.34 times. During a pandemic, it is important that the media work in coordination with the Ministry of Health and avoid spreading news that disconcerts society. Also, informing at-risk groups about their risks is important during a pandemic. Our study's limitations are that we performed it in a short time during a single influenza season and in a single healthcare center. Another limitation is that 2009 H1N1 vaccines started to be distributed among pregnant women only after the 2009 H1N1 epidemic obviously had started, so the low uptake rate might have resulted from the delay in vaccinations. As Schwarzinger et al. said, having influenza-like symptoms during that time could be associated with the vaccine's reduced acceptability in our study [23]. Further studies that substantiate our results should be performed with more pregnants in other hospitals and in other influenza seasons for to our results be generalized.

## Supporting Information

Table S1 Distribution of answer ‘yes’ given by 314 pregnant women concerning 2009 H1N1 vaccine.
(0.05 MB DOC)
