# Supplementary material for: Status of Pandemic Influenza Vaccination and Factors Affecting It in Pregnant Women in Kahramanmaras, an Eastern Mediterranean City of Turkey
Source: PLoS One. 2010 Dec 1;5(12):e14177. doi: 10.1371/journal.pone.0014177 (PMC2995732; doi:10.1371/journal.pone.0014177)
Supplement: Table S1 — Distribution of answer ‘yes’ given by 314 pregnant women concerning 2009 H1N1 vaccine. (0.05 MB DOC) [file pone.0014177.s001.doc]

**Table S1**. Distribution of answer ‘yes’ given by 314 pregnant women concerning 2009 H1N1 vaccine.

|  | **Yes** |
| --- | --- |
|  | **%** |
| **2009 H1N1 vaccine side effects** |  |
| Ruddiness, swelling, hardness, cyanosis, and pain can ocur at the site of the vaccination | 33.1 |
| Mercury in the vaccine causes cancer and death | 74.5 |
| The vaccine can cause lassitude and fatigue | 31.2 |
| The vaccine can cause headaches | 22.6 |
| The vaccine can cause apoplexy and neropathies | 28.0 |
| The vaccine can cause perspiration and tremors | 24.8 |
| The vaccine can cause joint and muscle aches | 24.8 |
| The vaccine can cause extensive skin reactions (including urticaria) | 25.5 |
| The vaccine can cause a reduction in tension | 23.2 |
| The vaccine can cause shock | 20.4 |
| The vaccine can cause pain throughout the nervous system | 18.8 |
| The vaccine can cause a decrease in blood clotting, which can lead to bleeding | 17.8 |
| The vaccine can cause vascularitis | 16.2 |
| The vaccine can cause Guillain-Barre Syndrome | 13.1 |
| The vaccine is harmful in the long term | 75.5 |
| **2009 H1N1 vaccine side effects related to pregnancy** |  |
| The vaccine can cause miscarriage | 70.1 |
| The vaccine can cause deformation in the child | 74.2 |
| The vaccine can cause infertility | 72.3 |
| **Beliefs about a mass H1N1 vaccination campaign conspiracy** |  |
| The 2009 H1N1 vaccine will be performed first and only in Turkey, and it will be tried on us as test subjects | 62.4 |
| All the 2009 H1N1 vaccines do not have approval | 69.1 |
| There are are some other countries manipulating the receipt of the 2009 H1N1 vaccine | 77.4 |
| The vaccine companies creating 2009 H1N1 | 77.1 |
| **Other attitudes and beliefs about 2009 H1N1 vaccine** |  |
| The vaccine protects 100% against the 2009 H1N1 | 66.9 |
| The 2009 H1N1 spreads much faster than the seasonal flu | 21.0 |
| The fatality of 2009 H1N1 is lower than that of the seasonal flu | 79.9 |
